# Activation Mechanism of LRRK2 and Its Cellular Functions in Parkinson's Disease

**DOI:** 10.1155/2016/7351985

**Published:** 2016-05-12

**Authors:** Katharina E. Rosenbusch, Arjan Kortholt

**Affiliations:** Department of Cell Biochemistry, University of Groningen, Nijenborgh 7, 9747 AG Groningen, Netherlands

## Abstract

Human LRRK2 (Leucine-Rich Repeat Kinase 2) has been associated with both familial and idiopathic Parkinson's disease (PD). Although several LRRK2 mediated pathways and interaction partners have been identified, the cellular functions of LRRK2 and LRRK2 mediated progression of PD are still only partially understood. LRRK2 belongs to the group of Roco proteins which are characterized by the presence of a Ras-like G-domain (Roc), a C-terminal of Roc domain (COR), a kinase, and several protein-protein interaction domains. Roco proteins exhibit a complex activation mechanism involving intramolecular signaling, dimerization, and substrate/effector binding. Importantly, PD mutations in LRRK2 have been linked to a decreased GTPase and impaired kinase activity, thus providing putative therapeutic targets. To fully explore these potential targets it will be crucial to understand the function and identify the pathways responsible for LRRK2-linked PD. Here, we review the recent progress in elucidating the complex LRRK2 activation mechanism, describe the accumulating evidence that link LRRK2-mediated PD to mitochondrial dysfunction and aberrant autophagy, and discuss possible ways for therapeutically targeting LRRK2.

## 1. Introduction

Parkinson's disease (PD) is a progressive motor disorder that is caused by the degeneration of dopaminergic neurons in the midbrain. The prevalence of PD increases with age, with 2% of individuals over the age of 80 being affected thereby representing the second most common neurodegenerative disorder worldwide [1–3]. Causations are various and mostly divided into a sporadic form without a clear trigger and a familial form in which a genetic factor is involved. The monogenic form of PD is caused by a single mutation in a recessively or dominantly inherited gene. It has been found in sporadic as well as familial PD and accounts for approximately 3–5% and 30% of the cases, respectively [4, 5]. Mutations in SCNA and LRRK2 (Leucine-Rich Repeat Kinase 2) are a specific subset of familial PD as they are autosomal-dominant with LRRK2 representing the most common cause of inherited PD [5]. It belongs to the Roco family of proteins, which constitutes a novel family of Ras-like G-proteins being conserved in almost all kingdoms of life [6–8].

**Figure 1:**
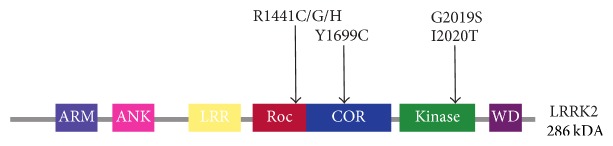
Schematic diagram of the domain architecture of LRRK2. Above, the segregating mutations of LRRK2 in Parkinson's disease are shown (arrows). ARM: Armadillo repeats, ANK: Ankyrin repeats, LRR: leucine-rich repeats, and WD: WD40 repeats.

LRRK2 is a large (286 kDa) and complex protein with a unique multiple-domain architecture (Figure 1), consisting of Armadillo repeats (ARM), Ankyrin repeats (ANK), leucine-rich repeats (LRR), a Ras of complex proteins (Roc), a C-terminal of Roc (COR), a kinase domain, and WD40 repeats [2, 6, 7].

Over 40 LRRK2 mutations have been identified representing risk factors for PD [9–11]. Most of the verified pathogenic PD-linked LRRK2 mutations are accumulated around the central core of the protein; one is found in the LRR, one in the Roc domain (with multiple substitutions), one in the COR domain, and two in the kinase domain (Figure 1). The multiple disease-linked mutations in LRRK2 represent a unique opportunity to explore the activation mechanism of the protein, its misregulation in PD, and the underlying molecular mechanisms of genetic and sporadic PD.

In this review, we will focus on the recent progress in elucidating the complex LRRK2 activation mechanism, highlight the evidence for a role of LRRK2 in the mitochondrial and autophagy pathways, and discuss possible ways to therapeutically target LRRK2-mediated PD.

## 2. LRRK2 Kinase and GTPase Activity

**Figure 2:**
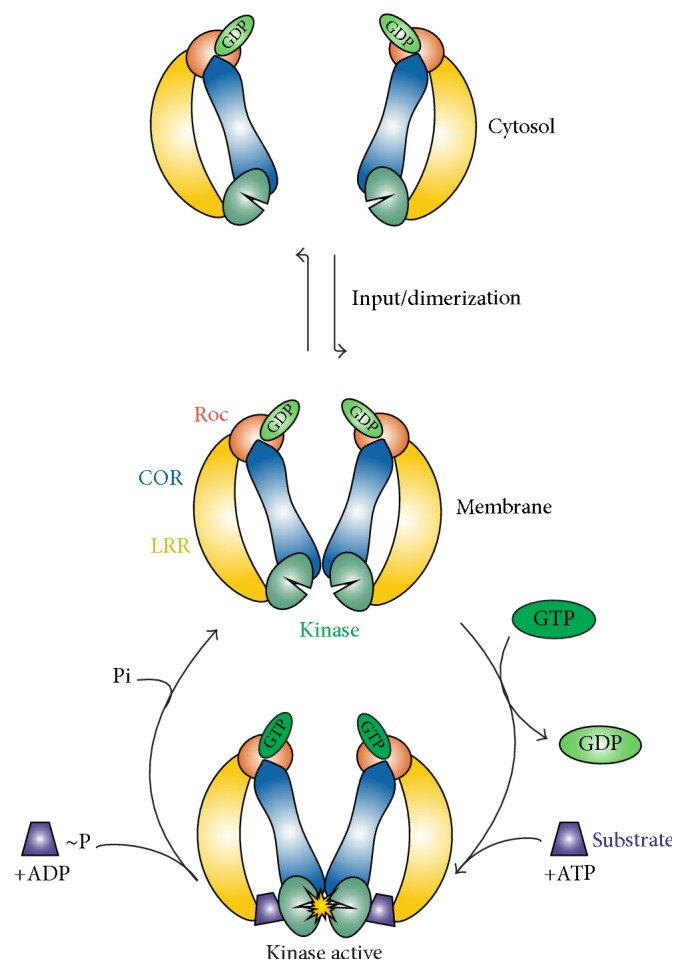
Proposed model of the activation mechanism of LRRK2. LRRK2 activation is at least regulated by three different mechanisms: cycling between (1) an almost inactive monomer and active dimer at the membrane, (2) intramolecular activation, and (3) binding of input/substrate to the N- and C-terminal domains.

LRRK2 has two bona fide enzymatic activities via its Roc (GTPase) and kinase domain. Several studies have shown that the Serine/Threonine specific kinase activity is responsible for LRRK2-mediated PD symptoms, including the degeneration of nigrostriatal dopaminergic neurons and the formation of Lewy bodies [2, 4, 12–14]. While PD-mutated LRRK2 triggers increased inclusion body formation in SH-SY5Y and cell death in primary rat cortical neurons, both of these phenotypes were diminished upon introduction of a LRRK2 kinase dead mutation [15]. For a long time, the function of the kinase domain has been considered as the main output of LRRK2. However, only for the G2019S PD mutation, representing the most common pathogenic point mutation, an increased phosphorylation activity has been reported [16–18]. For other pathogenic mutations, inconsistent, modest, or no effect on kinase activity has been shown [16–18]. Furthermore, PD mutations in LRRK2 probably have different defects in its activation mechanism and it is unclear if all pathogenic effects are mediated via the kinase domain [17–19]. Also the enzymatic activity of the Roc domain is affected in LRRK2-mediated PD-mutants and recent data strongly suggest that PD mutations in both Roc and COR domains result in decreased GTP hydrolysis [18, 20–24]. The Roc domain of LRRK2 belongs to the family of small G-proteins which are GTP binding proteins switching between an active GTP- and inactive GDP-bound state (Figure 2) [25]. Studies with both LRRK2 and an amoebic homologue revealed that a functional Roc domain is essential for kinase activity and disruption of Roc or the kinase domain by a single point mutation leads to the complete inactivation of the protein [15, 22, 23, 26].* In vivo* studies with LRRK2 G2019S showed that primary neurons possess a lower level of toxicity after the GTPase function was abolished [27]. Further studies confirmed that GTPase activity is central for neuronal toxicity and LRRK2 pathobiology in human cell lines and model organisms [20–22]. However, the data prove the involvement of both enzymatic activities in the onset of PD and imply a present cross-talk between the two domains.

## 3. LRRK2 Activation Mechanism

**Figure 3:**
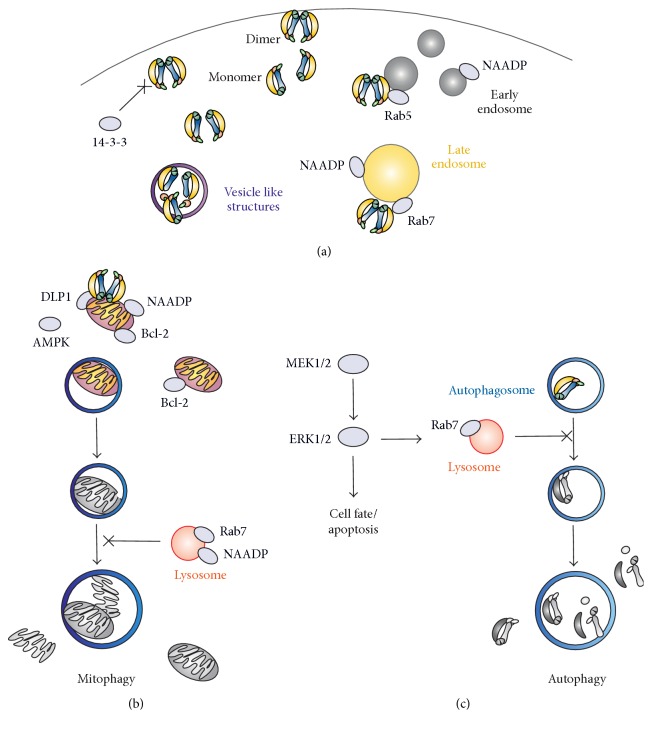
Proposed pathways regulating LRRK2-mediated mitochondrial homeostasis and autophagy.

The exact molecular mechanism by which the catalytic activity of LRRK2 is regulated remains unknown; however, accumulating evidence suggests the involvement of at least three different mechanisms: dimerization in close association with localization, intramolecular activation, and binding of input/substrate to the N- and C-terminal domains (Figures 2 and 3).

LRRK2 is monomeric and almost inactive in the cytosol, while it is predominantly dimeric and active when localized at the membrane [28–32]. Membrane enriched LRRK2 displayed an enhanced molecular mass as well as a 8.4 times higher kinase activity in comparison to the cytosolic LRRK2 suggesting that localization is dependent on and affects phosphorylation [31, 33, 34]. Structural studies with bacterial Roco proteins have revealed that the COR domain functions as an essential dimerization device [35]. During dimerization, the catalytic machinery for the GTPase reaction is being formed by complementation of the active site of one protomer with the other protomer [33, 35]. COR truncated proteins that are not able to dimerize have a drastically lower (700 times) GTPase activity. Interestingly, abolishing dimerization also alters autophosphorylation levels, indicating that both enzymatic activities are critically dependent on dimerization [35–38]. In this way, the intramolecular GTPase reaction functions as a timing device for the activation and biological functions of Roco proteins. Interestingly, a recent study has shown that mutations of known phosphorylation sides in the G-domain affect both kinase and GTPase activity [30]. Together the data suggest that the Roc-COR tandem is regulating kinase activity, the kinase is regulating the GTPase activity of Roc, and both events are critically involved in LRRK2 cellular distribution.

LRRK2 dimerization and activation is regulated by the N- and C-terminal LRRK2 protein-protein interaction domains. Cellular studies with LRRK2 and related Roco proteins lacking the N- or C-terminus suggested their essential role for signaling* in vivo* [7, 39].

Deletion of the WD40 repeats led to impaired dimer formation accompanied with diminished kinase activity and aberrant protein localization [40]. Recent data suggest that the N-terminus inhibits LRRK2 kinase activity, since deletion of the terminus resulted in increased LRRK2 autophosphorylation levels when expressed in human cell lines [32]. On the contrary, LRRK2 G2019S PD mutation displayed increased kinase activity with a lower level of autophosphorylation of the N-terminus (S910/935~P) [32, 41]. Although the N- and C-terminus of LRRK2 have an essential role* in vivo*, they are not required for kinase activity* in vitro* [7, 39]. This might suggest that the N- and C-terminal protein-protein interaction domains regulate LRRK2 activity by binding to upstream and/or downstream effectors. In this perspective, it has been shown that the N-terminal segment of LRRK2 interacts in a phosphorylation dependent manner with the ubiquitous regulatory protein 14-3-3. Disruption of the phosphorylation sides S910 and S935 blocks 14-3-3 binding and leads to the delocalization of LRRK2 from the membrane and its accumulation in the cytosol (Figure 3) [34, 41]. Recently, members of the Rab family of small GTPases have been identified as valid LRRK2 interactors and substrates [42–44].* In vivo* studies confirmed direct binding, most likely mediated via the N-terminus, and colocalization of LRRK2 with Rab5 and Rab7, suggesting an involvement in degradative and endocytic membrane trafficking (Figure 3). Strikingly, the PD mutation G2019S disrupted molecular trafficking and colocalization with Rab7, resulting in the formation of aberrant endosomal structures and endosomal/lysosomal localization thus interfering with the cellular degradative trafficking pathway of organelles [45, 46]. Furthermore, LRRK2 binding to Rab32 is regulating its localization to lysosomes as well as mitochondria [47].

## 4. LRRK2-Mediated Mitochondrial Dysfunction, Autophagy, and Cell Death

Numerous potential LRRK2 mediated pathways have been identified; however, much about its cellular functions and LRRK2 mediated progression of PD remains unknown. Accumulating evidence links LRRK2-mediated PD to mitochondrial dysfunction and aberrant autophagy (Figure 3) [48–51]. LRRK2 transfected HEK-293T cells showed a 10% enhanced localization of LRRK2 to the outer but not inner mitochondrial membrane [51]. The morphology and interconnectivity of mitochondria in skin samples of G2019S carrier patients were detected to be abnormal, most likely due to dysregulated fission and fusion events [50]. Analysis of the substantia nigra of patients with idiopathic PD revealed a glutathione depletion and mitochondrial complex-I deficiency, both representing known indicators of oxidative stress [52]. Furthermore, polymorphism in mtDNA (mitochondrial DNA) and aberrant levels of the neurotoxin MPP+ (1-methyl-4-phenylpyridinium) and its precursor MPTP (1-methyl-4-phenyl-1,2,3,6-tetrahydropyridine) were found in patient samples, various model organisms, and human culture cell lines [53]. In neurons it was shown that LRRK2 colocalizes with the Dynamin like protein 1 (DLP1), a known mitochondrial fission factor (Figure 3). Expression of LRRK2 G2019S and R1441C in neurons induced mitochondrial fragmentation and increased their interaction rate with DLP1 which also displayed higher phosphorylation levels, resulting, among others, in an enhanced level of reactive oxygen species (ROS). All these defects could be rescued by silencing of DLP1, suggesting a LRRK2/DLP1 pathway regulating mitochondrial fission events and their clearance [51, 54]. Localization of LRRK2 is not limited to mitochondrial structures but was found at a variety of additional membranes, including multivesicular bodies (MVBs) representing autophagic vacuoles (AVs) [55]. Consistently, being involved in the regulation of the endosomal-autophagic pathway, expression of PD-mutated LRRK2 triggered the accumulation of (abnormal) MVBs and AVs via misbalancing the induction of macroautophagy and maturation of AVs to lysosomes (Figure 3) [55]. Furthermore, expression of LRRK2 G2019S in human cell lines led to the shortening of neurite length and an increase in autophagic vacuole levels [15, 56].

The pathways regulating and linking LRRK2 PD-mediated mitochondrial dysregulation and abnormal autophagy are only partly identified but most likely include the activation of the autophagy regulating protein 5′ AMP-activated protein kinase (AMPK) [45, 57]. The abnormal kinase activity of LRRK2 G2019S in human cell lines leads to an increased level of phosphorylated AMPK, which subsequently results in enhanced levels of autophagosomes [58]. The mitogen-activated protein kinases (MAPK) cascade may represent another important pathway regulating LRRK2-mediated autophagy. In addition to enhanced autophagic activity and cell death, cells expressing LRRK2 G2019S also showed a threefold increase in protein turnover and a higher level of phosphorylated MAPK/ERK. Incubation with a specific inhibitor of MEK1/2 (U0126) was sufficient to rescue the aberrant phenotypes of the LRRK2 G2019S cells [56, 59]. It was suggested that LRRK2 induces autophagy via the activation of NAADP (nicotinic acid adenine dinucleotide phosphate) receptors, which are involved in the calcium efflux from endosomes [58]. The mitochondrial antiapoptotic protein, Bcl-2, might represent the connection between LRRK2-induced dysregulated mitochondrial homeostasis and autophagy. Expression of phosphorylated Bcl-2 rescues both the mitochondrial and autophagy defects of LRRK2 G2019S cells [60].

Several other PD associated proteins, including *α*-synuclein, Parkin, DJ-1, PINK1, and HtrA2, have been linked to similar defects in mitochondria regulation and autophagy [37, 61, 62]. Parkin, a known regulator of mitochondrial clearance, and AMPK seem to be directly involved in an alternative or parallel pathway as overexpression acted protectively against cellular toxicity in fly dopaminergic neurons expressing mutated LRRK2 [61]. Mutations in Parkin and PINK1 (PTEN-induced kinase 1), both mitochondria regulating proteins, have been found in sporadic as well as autosomal recessive PD and result in severe mitochondrial abnormalities and cell death [63]. The parallel expression of PD-LRRK2 in PINK1 and DJ-1 deficient fly cells or mice neurons with abnormal *α*-synuclein activity leads to an increase of respective pathogenic phenotypes [64, 65]. Deletion of LRRK2 acts in a neuroprotective way towards *α*-synuclein mediated effects in mouse models [65]. DJ-1 is only partially able to rescue the phenotypes of PINK1 mutated neurons but, vice versa, overexpression of both Parkin and PINK1 restores the abnormal mitochondrial morphologies of DJ-1 deficient cells, suggesting a present connection between the involved pathways [66, 67]. Altogether it might suggest the presence of common PD-pathogenic pathways that result in mitochondrial dysfunction and autophagy.

## 5. Therapeutic Targeting of LRRK2

The major focus of academia and industry is the development of kinase inhibitors as potential therapeutics for LRRK2-mediated PD. Almost all clinical kinase inhibitors are used for short-time treatment in the cancer field and for immunological, neurological, and infectious diseases, where side effects caused by high dosage are tolerated [68]. In contrast, for the long-term treatment of chronic diseases such as LRRK2-associated PD no potential toxic side effects can be present. Several highly specific and brain penetrant LRRK2 kinase inhibitors were identified but have yet to be optimized in order to qualify as drug candidates for therapeutic treatment [69–72]. Our structures of a humanized* D. discoideum* Roco4 kinase domain bound to the common inhibitors LRRK2-IN-1 or Compound 19 revealed a highly similar binding mechanism and gave important information for potential optimization [73]. However, accumulation in peripheral tissues, especially kidneys and lungs, and related drug induced toxicity are still a major and common problem for all LRRK2 kinase inhibitors [72, 74, 75]. In rodent models, enhanced dosages of the recent highly specific and brain penetrant LRRK2 kinase inhibitors GNE-7915 and GNE-0877 are well tolerated over a longer time period; however, they induced the cytoplasmic accumulation of lysosome-related organelles in the lungs of nonhuman primates [76].

Understanding how other domains of LRRK2 modulate its activity is an important but rather neglected field in LRRK2 research and not a focus of industries. However, the PD causing mutations are found in nearly all domains of LRRK2 leading to the same well described symptoms. Furthermore, as recent data suggest that different PD mutations have diverse defects with regard to the activation mechanism, they might require specified ways of inhibition for the purpose of drug development [72, 74, 77].

Alternative approaches targeting further LRRK2 domains and sites of its complex activation mechanism, including the N- and C-terminus, the catalytic GTPase activity of Roc, LRRK2 localization, dimerization, or allosteric modulation of the kinase domain, might significantly improve therapeutic benefits (Figure 2).

The LRRK2 mutations in the Roc (R1441C/G/H) and COR (Y1699C) domain have a decreased GTPase activity and a functional LRRK2 G-domain is essential for LRRK2 activation, suggesting GTPase activity forms an interesting therapeutic target [20, 78–80]. Targeting the G-domain could be done by using small compounds that bind and interfere with nucleotide binding, resemble the GDP-bound off-state, or increase the GTPase cycle. Recently, the first LRRK2 GTP binding inhibitors, compounds 68 and 70, were identified and proved to inhibit both GTPase and kinase activity* in vitro* as well as* in vivo* and thereby attenuated neuronal degeneration in human cell lines/rodent tissues [14]. Importantly, FX2149, a novel analog of 68, even displayed an around two times higher brain inhibition efficiency in a rodent model organism [81].

The N- and C-terminal segments of LRRK2 contain several protein-protein interaction domains which are involved in regulating kinase activity, oligomerization, and/or localization. As described above, LRRK2 cycles between a low active monomeric cytosolic state and a high active dimeric membrane bound state. Importantly, since LRRK2 activation is dependent on membrane localization and dimerization, inhibiting either of these properties may be a good therapeutic approach.

## 6. Summary

Recent studies have shed light on the complex activation mechanism of LRRK2 and revealed highly precise and exact timed interactions on both intra- and intermolecular levels. These multiple layers of regulation and enzyme activities within one protein make LRRK2 an interesting therapeutic target. To further explore these therapeutic targets, it will be essential to completely characterize the molecular activation mechanism. Biochemical and structural characterization of LRRK2 and/or related Roco proteins can give important information about the dimerization mechanism, how the kinase domain regulates GTPase activity, how LRRK2 activity is regulated by binding of input or substrate to the LRR and WD40 domains, and how the PD mutations influence the complex regulatory mechanism. Recent data strongly suggest that LRRK2 dysfunction in PD results in mitochondrial defects and autophagy. However, the precise underlying mechanisms are still not well understood and many questions about the cellular function of LRRK2 remain to be addressed, including at which (inter)cellular membrane LRRK2 is activated and if common underlying pathways of familial PD are existing. To answer these questions, it will be crucial to identify physiological kinase substrate(s) and upstream and downstream regulators.

## References

[1] Goetz C. G. (2011). The history of Parkinson's disease: early clinical descriptions and neurological therapies. *Cold Spring Harbor Perspectives in Medicine*.

[2] Dächsel J. C., Farrer M. J. (2010). LRRK2 and Parkinson disease. *Archives of Neurology*.

[3] Lees A. J., Hardy J., Revesz T. (2009). Parkinson's disease. *The Lancet*.

[4] Gilks W. P., Abou-Sleiman P. M., Gandhi S. (2005). A common LRRK2 mutation in idiopathic Parkinson's disease. *The Lancet*.

[5] Klein C., Westenberger A. (2012). Genetics of Parkinson's disease. *Cold Spring Harbor Perspectives in Medicine*.

[6] Marín I., van Egmond W. N., van Haastert P. J. M. (2008). The Roco protein family: a functional perspective. *The FASEB Journal*.

[7] van Egmond W. N., van Haastert P. J. M. (2010). Characterization of the Roco protein family in *Dictyostelium discoideum*. *Eukaryotic Cell*.

[8] Bosgraaf L., Van Haastert P. J. M. (2003). Roc, a Ras/GTPase domain in complex proteins. *Biochimica et Biophysica Acta (BBA)—Molecular Cell Research*.

[9] Deng H., Le W., Guo Y. (2006). Genetic analysis of LRRK2 mutations in patients with Parkinson disease. *Journal of the Neurological Sciences*.

[10] Trabzuni D., Ryten M., Emmett W. (2013). Fine-mapping, gene expression and splicing analysis of the disease associated LRRK2 locus. *PLoS ONE*.

[11] Bonifati V. (2014). Parkinsonism and related disorders genetics of Parkinson's disease—state of the art, 2013. *Parkinsonism & Related Disorders*.

[12] Thomas B., Beal M. F. (2007). Parkinson's disease. *Human Molecular Genetics*.

[13] Zimprich A., Biskup S., Leitner P. (2004). Mutations in LRRK2 cause autosomal-dominant parkinsonism with pleomorphic pathology. *Neuron*.

[14] Li T., Yang D., Zhong S. (2014). Novel LRRK2 GTP-binding inhibitors reduced degeneration in Parkinson's disease cell and mouse models. *Human Molecular Genetics*.

[15] Greggio E., Jain S., Kingsbury A. (2006). Kinase activity is required for the toxic effects of mutant LRRK2/dardarin. *Neurobiology of Disease*.

[16] Anand V. S., Braithwaite S. P. (2009). LRRK2 in Parkinson's disease: biochemical functions. *FEBS Journal*.

[17] Cookson M. R. (2010). The role of leucine-rich repeat kinase 2 (LRRK2) in Parkinson's disease. *Nature Reviews Neuroscience*.

[18] Esteves A. R., Swerdlow R. H., Cardoso S. M. (2014). LRRK2, a puzzling protein: insights into Parkinson's disease pathogenesis. *Experimental Neurology*.

[19] Mills R. D., Mulhern T. D., Liu F., Culvenor J. G., Cheng H.-C. (2014). Prediction of the repeat domain structures and impact of parkinsonism-associated variations on structure and function of all functional domains of leucine-rich repeat kinase 2 (LRRK2). *Human Mutation*.

[20] Biosa A., Trancikova A., Civiero L. (2013). GTPase activity regulates kinase activity and cellular phenotypes of parkinson's disease-associated LRRK2. *Human Molecular Genetics*.

[21] Stafa K., Trancikova A., Webber P. J., Glauser L., West A. B., Moore D. J. (2012). GTPase activity and neuronal toxicity of Parkinson's disease-associated LRRK2 is regulated by ArfGAP1. *PLoS Genetics*.

[22] Xiong Y., Coombes C. E., Kilaru A. (2010). GTPase activity plays a key role in the pathobiology of LRRK2. *PLoS Genetics*.

[23] Ito G., Okai T., Fujino G. (2007). GTP binding is essential to the protein kinase activity of LRRK2, a causative gene product for familial Parkinson's disease. *Biochemistry*.

[24] West A. B., Moore D. J., Choi C. (2007). Parkinson's disease-associated mutations in LRRK2 link enhanced GTP-binding and kinase activities to neuronal toxicity. *Human Molecular Genetics*.

[25] Vetter I. R., Wittinghofer A. (2001). The guanine nucleotide-binding switch in three dimensions. *Science*.

[26] Taymans J.-M., Vancraenenbroeck R., Ollikainen P. (2011). LRRK2 kinase activity is dependent on LRRK2 gtp binding capacity but independent of LRRK2 GTP binding. *PLoS ONE*.

[27] Smith W. W., Pei Z., Jiang H., Dawson V. L., Dawson T. M., Ross C. A. (2006). Kinase activity of mutant LRRK2 mediates neuronal toxicity. *Nature Neuroscience*.

[28] James N. G., Digman M. A., Gratton E. (2012). Number and brightness analysis of LRRK2 oligomerization in live cells. *Biophysical Journal*.

[29] Schapansky J., Nardozzi J. D., Felizia F., LaVoie M. J. (2014). Membrane recruitment of endogenous LRRK2 precedes its potent regulation of autophagy. *Human Molecular Genetics*.

[30] Webber P. J., Smith A. D., Sen S., Renfrow M. B., Mobley J. A., West A. B. (2011). Autophosphorylation in the leucine-rich repeat kinase 2 (LRRK2) GTPase domain modifies kinase and GTP-binding activities. *Journal of Molecular Biology*.

[31] Berger Z., Smith K. A., Lavoie M. J. (2010). Membrane localization of LRRK2 is associated with increased formation of the highly active lrrk2 dimer and changes in its phosphorylation. *Biochemistry*.

[32] Greggio E., Zambrano I., Kaganovich A. (2008). The Parkinson disease-associated leucine-rich repeat kinase 2 (LRRK2) is a dimer that undergoes intramolecular autophosphorylation. *The Journal of Biological Chemistry*.

[33] Sen S., Webber P. J., West A. B. (2009). Dependence of leucine-rich repeat kinase 2 (LRRK2) kinase activity on dimerization. *The Journal of Biological Chemistry*.

[34] Nichols R. J., Dzamko N., Morrice N. A. (2010). 14-3-3 Binding to LRRK2 is disrupted by multiple Parkinson's disease-associated mutations and regulates cytoplasmic localization. *Biochemical Journal*.

[35] Gotthardt K., Weyand M., Kortholt A., Van Haastert P. J. M., Wittinghofer A. (2008). Structure of the Roc-COR domain tandem of *C. tepidum*, a prokaryotic homologue of the human LRRK2 Parkinson kinase. *The EMBO Journal*.

[36] Greggio E., Taymans J.-M., Zhen E. Y. (2009). The Parkinson's disease kinase LRRK2 autophosphorylates its GTPase domain at multiple sites. *Biochemical and Biophysical Research Communications*.

[37] Deng J., Lewis P. A., Greggio E., Sluch E., Beilina A., Cookson M. R. (2008). Structure of the ROC domain from the Parkinson's disease-associated leucine-rich repeat kinase 2 reveals a dimeric GTPase. *Proceedings of the National Academy of Sciences of the United States of America*.

[38] Liao J., Wu C.-X., Burlak C. (2014). Parkinson disease-associated mutation R1441H in LRRK2 prolongs the ‘active state’ of its GTPase domain. *Proceedings of the National Academy of Sciences of the United States of America*.

[39] Iaccarino C., Crosio C., Vitale C., Sanna G., Carrì M. T., Barone P. (2007). Apoptotic mechanisms in mutant LRRK2-mediated cell death. *Human Molecular Genetics*.

[40] Jorgensen N. D., Peng Y., Ho C. C.-Y. (2009). The WD40 domain is required for LRRK2 neurotoxicity. *PLoS ONE*.

[41] Reynolds A., Doggett E. A., Riddle S. M., Lebakken C. S., Nichols R. J. (2014). LRRK2 kinase activity and biology are not uniformly predicted by its autophosphorylation and cellular phosphorylation site status. *Frontiers in Molecular Neuroscience*.

[42] Novick P., Zerial M. (1997). The diversity of Rab proteins in vesicle transport. *Current Opinion in Cell Biology*.

[43] Beilina A., Rudenko I. N., Kaganovich A. (2014). Unbiased screen for interactors of leucine-rich repeat kinase 2 supports a common pathway for sporadic and familial Parkinson disease. *Proceedings of the National Academy of Sciences of the United States of America*.

[44] Steger M., Tonelli F., Ito G. (2016). Phosphoproteomics reveals that Parkinson's disease kinase LRRK2 regulates a subset of Rab GTPases. *eLife*.

[45] Gómez-Suaga P., Rivero-Ríos P., Fdez E. (2014). LRRK2 delays degradative receptor trafficking by impeding late endosomal budding through decreasing Rab7 activity. *Human Molecular Genetics*.

[46] Dodson M. W., Zhang T., Jiang C., Chen S., Guo M. (2012). Roles of the *Drosophila LRRK2* homolog in Rab7-dependent lysosomal positioning. *Human Molecular Genetics*.

[47] Waschbüsch D., Michels H., Strassheim S. (2014). LRRK2 transport is regulated by its novel interacting partner Rab32. *PLoS ONE*.

[48] Biskup S., Moore D. J., Celsi F. (2006). Localization of LRRK2 to membranous and vesicular structures in mammalian brain. *Annals of Neurology*.

[49] Schapira A. H. V., Cooper J. M., Dexter D., Clark J. B., Jenner P., Marsden C. D. (1990). Mitochondrial complex I deficiency in Parkinson's disease. *Journal of Neurochemistry*.

[50] Mortiboys H., Johansen K. K., Aasly J. O., Bandmann O. (2010). Mitochondrial impairment in patients with Parkinson disease with the G2019S mutation in LRRK2. *Neurology*.

[51] West A. B., Moore D. J., Biskup S. (2005). Parkinson's disease-associated mutations in leucine-rich repeat kinase 2 augment kinase activity. *Proceedings of the National Academy of Sciences of the United States of America*.

[52] Bénit P., Lebon S., Chol M., Giurgea I., Rötig A., Rustin P. (2004). Mitochondrial complex I deficiency in humans. *Current Genomics*.

[53] Lin M. T., Beal M. F. (2006). Mitochondrial dysfunction and oxidative stress in neurodegenerative diseases. *Nature*.

[54] Niu J., Yu M., Wang C., Xu Z. (2012). Leucine-rich repeat kinase 2 disturbs mitochondrial dynamics via dynamin-like protein. *Journal of Neurochemistry*.

[55] Alegre-Abarrategui J., Christian H., Lufino M. M. P. (2009). LRRK2 regulates autophagic activity and localizes to specific membrane microdomains in a novel human genomic reporter cellular model. *Human Molecular Genetics*.

[56] Sambasivarao S. V. (2013). Role of autophagy in G2019S-LRRK2-associated neurite shortening in differentiated SH-SY5Y cells. *Journal of Neurochemistry*.

[57] Papkovskaia T. D., Chau K.-Y., Inesta-Vaquera F. (2012). G2019s leucine-rich repeat kinase 2 causes uncoupling protein-mediated mitochondrial depolarization. *Human Molecular Genetics*.

[58] Gómez-Suaga P., Luzón-Toro B., Churamani D. (2012). Leucine-rich repeat kinase 2 regulates autophagy through a calcium-dependent pathway involving NAADP. *Human Molecular Genetics*.

[59] Bravo-San Pedro J. M., Niso-Santano M., Gómez-Sánchez R. (2013). The LRRK2 G2019S mutant exacerbates basal autophagy through activation of the MEK/ERK pathway. *Cellular and Molecular Life Sciences*.

[60] Su Y.-C., Guo X., Qi X. (2015). Threonine 56 phosphorylation of Bcl-2 is required for LRRK2 G2019S-induced mitochondrial depolarization and autophagy. *Biochimica et Biophysica Acta (BBA)—Molecular Basis of Disease*.

[61] Hang L., Thundyil J., Lim K.-L. (2015). Mitochondrial dysfunction and Parkinson disease: a Parkin-AMPK alliance in neuroprotection. *Annals of the New York Academy of Sciences*.

[62] Vande Walle L., Lamkanfi M., Vandenabeele P. (2008). The mitochondrial serine protease HtrA2/Omi: an overview. *Cell Death and Differentiation*.

[63] Deng H., Dodson M. W., Huang H., Guo M. (2008). The Parkinson's disease genes *pink1* and *parkin* promote mitochondrial fission and/or inhibit fusion in *Drosophila*. *Proceedings of the National Academy of Sciences of the United States of America*.

[64] Venderova K., Kabbach G., Abdel-Messih E. (2009). Leucine-rich repeat kinase 2 interacts with Parkin, DJ-1 and PINK-1 in a *Drosophila melanogaster* model of Parkinson's disease. *Human Molecular Genetics*.

[65] Lin X., Parisiadou L., Gu X.-L. (2009). Leucine-rich repeat kinase 2 regulates the progression of neuropathology induced by Parkinson's-disease-related mutant *α*-synuclein. *Neuron*.

[66] Irrcher I., Aleyasin H., Seifert E. L. (2010). Loss of the Parkinson's disease-linked gene DJ-1 perturbs mitochondrial dynamics. *Human Molecular Genetics*.

[67] Thomas K. J., McCoy M. K., Blackinton J. (2011). DJ-1 acts in parallel to the PINK1/parkin pathway to control mitochondrial function and autophagy. *Human Molecular Genetics*.

[68] Cohen P. (2002). Protein kinases—the major drug targets of the twenty-first century?. *Nature Reviews Drug Discovery*.

[69] Reith A. D., Bamborough P., Jandu K. (2012). GSK2578215A; a potent and highly selective 2-arylmethyloxy-5-substitutent-*N*-arylbenzamide LRRK2 kinase inhibitor. *Bioorganic and Medicinal Chemistry Letters*.

[70] Ramsden N., Perrin J., Ren Z. (2011). Chemoproteomics-based design of potent LRRK2-selective lead compounds that attenuate Parkinson's disease-related toxicity in human neurons. *ACS Chemical Biology*.

[71] Choi H. G., Zhang J., Deng X. (2012). Brain penetrant LRRK2 inhibitor. *ACS Medicinal Chemistry Letters*.

[72] Deng X., Dzamko N., Prescott A. (2011). Characterization of a selective inhibitor of the Parkinson's disease kinase LRRK2. *Nature Chemical Biology*.

[73] Gilsbach B. K., Messias A. C., Ito G. (2015). Structural characterization of LRRK2 inhibitors. *Journal of Medicinal Chemistry*.

[74] Deng X., Choi H. G., Buhrlage S. J., Gray N. S. (2012). Leucine-rich repeat kinase 2 inhibitors: a patent review (2006–2011). *Expert Opinion on Therapeutic Patents*.

[75] Estrada A. A., Sweeney Z. K. (2015). Chemical biology of leucine-rich repeat kinase 2 (LRRK2) inhibitors. *Journal of Medicinal Chemistry*.

[76] Fuji R. N., Flagella M., Baca M. (2015). Effect of selective LRRK2 kinase inhibition on nonhuman primate lung. *Science Translational Medicine*.

[77] Jaleel M., Nichols R. J., Deak M. (2007). LRRK2 phosphorylates moesin at threonine-558: characterization of how Parkinson's disease mutants affect kinase activity. *Biochemical Journal*.

[78] Taymans J.-M. (2012). The GTPase function of LRRK2. *Biochemical Society Transactions*.

[79] Lewis P. A., Greggio E., Beilina A., Jain S., Baker A., Cookson M. R. (2007). The R1441C mutation of LRRK2 disrupts GTP hydrolysis. *Biochemical and Biophysical Research Communications*.

[80] Li X., Tan Y.-C., Poulose S., Olanow C. W., Huang X.-Y., Yue Z. (2007). Leucine-rich repeat kinase 2 (LRRK2)/PARK8 possesses GTPase activity that is altered in familial Parkinson's disease R1441C/G mutants. *Journal of Neurochemistry*.

[81] Li T., He X., Thomas J. M. (2015). A novel GTP-binding inhibitor, FX2149, attenuates LRRK2 toxicity in Parkinson's disease models. *PLoS ONE*.

